# Pseudoarthrosis of tibia and fibula

**DOI:** 10.11604/pamj.2023.44.112.39077

**Published:** 2023-03-01

**Authors:** Suyash Yashwant Ambatkar, Megha Dipak Rudey

**Affiliations:** 1 Department of Orthopedics, Datta Meghe Institute of Medical Sciences (DU), Sawangi, Wardha, Maharashtra, India,; 2 Department of Kaumarbhritya, Mahatma Gandhi Ayurveda College, Hospital and Research Centre, Salod (H), Datta Meghe Institute of Medical Sciences (DU), Sawangi, Wardha, India

**Keywords:** Congenital, false joint, pathological fracture, pseudoarthrosis of tibia and fibula

## Image in medicine

Pseudoarthrosis can be defined as a disorder of the diaphysis which is revealed by either pseudarthrosis at birth or by a pathological fracture presenting in bone with modifications such as bowing, narrowing of the medullary canal. Congenital pseudarthrosis of the tibia (CPT) is a rare disease in children, with an estimated frequency of 1/150,000 births. Clinical presentations range from simple anterolateral tibial angulation to complete non-union with extensive bone defects. Normally CPT is unilateral, located at the junction of the middle and distal thirds of the tibial segment with no predominance for sex. A 9-year-old male child was brought to the orthopaedics outpatient department (OPD) with complaints of severe pain in his right leg with a history of fall from bed. Immediately after which his mother noticed a deformity in his right leg. X-ray of right leg anteroposterior view (AP) and lateral view was done which showed fracture, cupping of the proximal fragment of bone proximal to the fracture site with narrow appearance of the distal bone.

**Figure 1:**
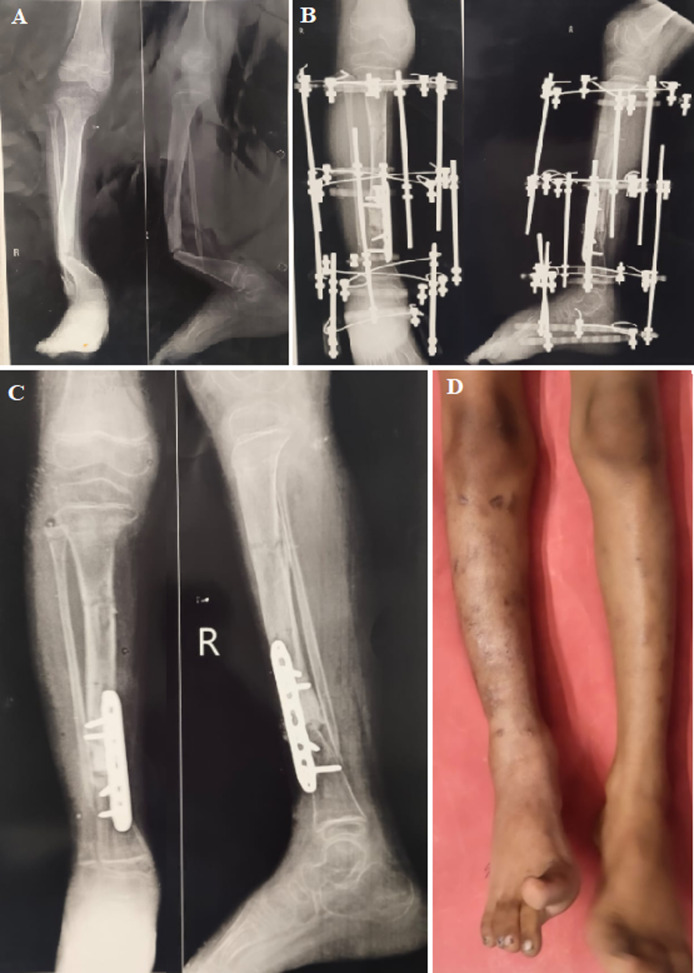
A) fracture of distal third tibia and fibula with cupping of the proximal fragment of, narrow appearance of the distal bone; B) post-operative X-ray showing fracture managed with Ilizarov ring fixator application and open reduction internal fixation with plate osteosynthesis; C) X-ray done after removal of the Ilizarov ring fixator showing union at the fracture site 2 months after the surgery; D) clinical photo of the leg showing corrected deformity

